# Explainable machine learning model reveals its decision-making process in identifying patients with paroxysmal atrial fibrillation at high risk for recurrence after catheter ablation

**DOI:** 10.1186/s12872-023-03087-0

**Published:** 2023-02-17

**Authors:** Yibo Ma, Dong Zhang, Jian Xu, Huani Pang, Miaoyang Hu, Jie Li, Shiqiang Zhou, Lanyan Guo, Fu Yi

**Affiliations:** Department of Cardiology, Xijing Hospital, Air Force Medical University, 169 Changle West Road, Xi’an, 710032 Shaanxi China

**Keywords:** Atrial fibrillation, Machine learning, Shapley additive explanations, Catheter ablation, Random forest

## Abstract

**Background:**

A number of models have been reported for predicting atrial fibrillation (AF) recurrence after catheter ablation. Although many machine learning (ML) models were developed among them, black-box effect existed widely. It was always difficult to explain how variables affect model output. We sought to implement an explainable ML model and then reveal its decision-making process in identifying patients with paroxysmal AF at high risk for recurrence after catheter ablation.

**Methods:**

Between January 2018 and December 2020, 471 consecutive patients with paroxysmal AF who had their first catheter ablation procedure were retrospectively enrolled. Patients were randomly assigned into training cohort (70%) and testing cohort (30%). The explainable ML model based on Random Forest (RF) algorithm was developed and modified on training cohort, and tested on testing cohort. In order to gain insight into the association between observed values and model output, Shapley additive explanations (SHAP) analysis was used to visualize the ML model.

**Results:**

In this cohort, 135 patients experienced tachycardias recurrences. With hyperparameters adjusted, the ML model predicted AF recurrence with an area under the curve of 66.7% in the testing cohort. Summary plots listed the top 15 features in descending order and preliminary showed the association between features and outcome prediction. Early recurrence of AF showed the most positive impact on model output. Dependence plots combined with force plots showed the impact of single feature on model output, and helped determine high risk cut-off points. The thresholds of CHA_2_DS_2_-VASc score, systolic blood pressure, AF duration, HAS-BLED score, left atrial diameter and age were 2, 130 mmHg, 48 months, 2, 40 mm and 70 years, respectively. Decision plot recognized significant outliers.

**Conclusion:**

An explainable ML model effectively revealed its decision-making process in identifying patients with paroxysmal atrial fibrillation at high risk for recurrence after catheter ablation by listing important features, showing the impact of every feature on model output, determining appropriate thresholds and identifying significant outliers. Physicians can combine model output, visualization of model and clinical experience to make better decision.

**Supplementary Information:**

The online version contains supplementary material available at 10.1186/s12872-023-03087-0.

## Introduction

One of the primary treatment options for atrial fibrillation (AF) is rhythm control. Catheter ablation is the first-line treatment for drug-refractory paroxysmal AF, and pulmonary vein isolation, the cornerstone of AF ablation, eradicates approximately 90% of AF triggers in principle [1, 2]. In fact, according to a meta-analysis on catheter ablation as the first-line treatment for paroxysmal AF, nearly 35% of patients will experience AF recurrence after the procedure [3]. Overall though the recurrence rate remains relatively high. This may weaken the confidence of patients in receiving rhythm control therapy, and lead to unfavorable outcomes such as persistent or permanent AF, heart failure, thromboembolism, dementia and so on. As a result, identifying the risk factors leading to AF recurrence is a crucial step.

Numerous studies have proposed a variety of prediction models, such as the HATCH, APPLE, or CAAP-AF score; however, the results differ greatly [4–6]. Collinearity exists in multiple clinical characteristics. Additionally, for univariate and multivariate regression analysis, the number of positive cases must be greater than about ten times the number of variables. Conventional statistical methods may be unable to handle such high-dimensionality data in the presence of too many variables. Consequently, many limitations may exist in the case of conventional statistical methods.

The use of machine learning (ML) algorithms in medicine has been gaining popularity and is helping physicians in clinical decision-making. ML algorithms can learn the association between multiple patient variables and clinical outcomes automatically. A large number of ML algorithms are non-parametric and are not limited by variable collinearity. Moreover, ML algorithms are appropriate for processing high-dimensionality data, given reasonable optimization [7]. Classification models based on ML, deep learning (DL) or radiomic methods to predict the outcome of AF have been reported in numerous studies [8–15]. Although the black-box property is common in ML models, interpretable methods can be used to understand their decision-making process and discover more potential information [16]. The aim of this study is to develop an explainable ML model based on a single center cohort as a proof-of-concept in order to reveal its decision-making process in identifying patients with paroxysmal AF at high risk for recurrence after catheter ablation.

## Methods

### Study design and population

A consecutive cohort of patients was included in the study from the First Affiliated Hospital of Air Force Medical University between January 2018 and December 2020. Patients over 18 years old with drug-refractory paroxysmal AF, who received their first catheter ablation procedure were eligible. Exclusion criteria included: (i) persistent, long-standing persistent or permanent AF; (ii) valvular AF, which was defined as AF occurring with moderate or severe mitral stenosis or surgical valve replacement; (iii) AF with primary cardiomyopathy (e.g., Hypertrophic Cardiomyopathy); (iv) reversible AF (e.g., AF associated with hyperthyroidism); (v) suspected compensated AF because of Sick Sinus Syndrome; (vi) patients who failed to follow-up. The electronic medical record system of the Information Department of the hospital showed 471 patients who met the criteria in the above period and were thus enrolled in the study. This study conformed to Transparent Reporting of a Multivariable Prediction Model for Individual Prognosis or Diagnosis (TRIPOD) statement and was approved by the ethics committee of the hospital, according to the principles of the Declaration of Helsinki. Written informed consent was acquired by all 471 patients before radiofrequency or cryoballoon ablation.

### Data collection

**Fig. 1:**
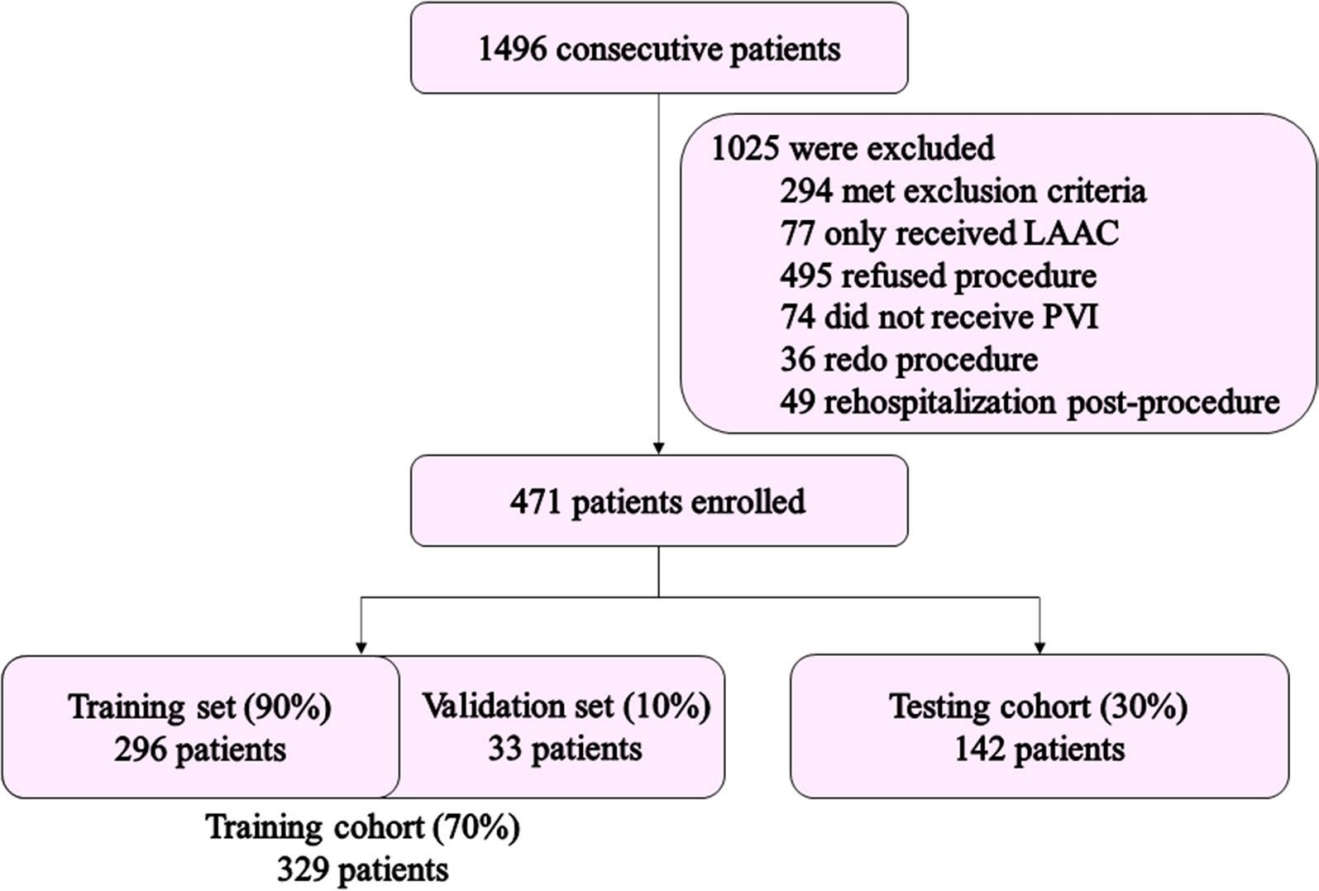
Study flowchart. LAAC left atrial appendage closure, PVI, pulmonary vein isolation

With "Atrial Fibrillation" as the search term, all patients hospitalized in the department of cardiovascular medicine from January 2018 to December 2020 were retrieved, and basic information of patients, including age, sex and ID number, were collected. Retrieval logic written in R language was used to automatically retrieve other relevant information such as medical history, physical examination, echocardiogram indexes, laboratory examination, procedural recordings, discharge medication and follow-up records, from the medical record management system. Basic information and data extracted by the retrieval logic were reviewed by two expert physicians to ensure accuracy. Patients were excluded based on the joint consensus of the two physicians if their information did not meet the criteria. The data collection flowchart is shown in Fig. 1.

### Feature selection

To ensure the robustness of the ML classification model, features were selected if they: (i) characterized the cardiac function, risk of systemic embolism or the level of inflammation, or (ii) promoted the occurrence and development of AF. Laboratory and echocardiographic characteristics were considered as missing values if they were not collected four weeks prior to the ablation procedure. Characteristics with more than 15% of missing values were excluded. Finally, 30 features were selected. Details of the features are presented in Additional file 1: Table S1.

### End point and follow-up

Recurrence of AF post-procedure was defined as the first episode of any type of atrial tachycardia, including but not limited to atrial flutter and AF, sustained for more than 30 s after the blanking period (BP). BP refers to the first three months post-procedure. In this period, any occurrence of atrial tachycardia was identified as early recurrence of AF (ERAF), which may relate to inflammatory response induced by electrical burn or freezing injury, rather than a true relapse [17]. The day of repeat ablation procedure was alternative if the patient could not remember when tachycardias recurred.

After the catheter ablation procedure, the outpatient follow-up was scheduled at one, three, six and every six months thereafter. At each visit, atrial tachycardia episodes were captured and confirmed by 12-lead electrocardiogram, Holter or ambulatory monitors. Communication with patients was established through online or telephone follow-up to ensure timely feedback on patients or inquire about the control of AF.

### Explainable ML model

The Random Forest (RF) algorithm was used in the building of an explainable ML model.

### Classifier illustration

RF is an ensemble algorithm based on the Decision Tree (DT). The basic idea of RF is bagging: features selected into the RF model are simultaneously voted by all independent DTs in the forest, and this process conforms to the principle of majority subordination. RF integrates the results of all DTs, obtaining higher model performance than any individual DT, while avoiding overfitting. At the same time, this algorithm can effectively solve the problem of feature collinearity because of its “if…else” calculation logic [18].

### Model development, validation and testing

The median of each feature was used to fill in the missing values, preserving the distribution of data to the greatest extent. However, underestimation of the weight of features using this method is possible. All patients and their specific characteristics in this cohort were randomly divided into the training and testing cohort (70% and 30% of the dataset, respectively). Because most of the hyperparameters are overfitted by default, a validation set, as an internal testing cohort, was needed to optimize the hyperparameters to ensure the better generalization of the ML model. This method enabled the loss reduction, model modification and determination of the weight of every selected feature. Therefore, the training cohort was randomly split into a training set and validation set in the ratio of 9:1. A tenfold cross validation method was used to evaluate the accuracy of the model in the training cohort to optimize the hyperparameters. The split of the datasets is shown in Fig. 1.

The optimal hyperparameters based on accuracy were determined step by step: first, learning curves were plotted to select the appropriate number of DTs, which may effectively reduce calculation time and avoid introducing too many systematic errors; second, repeated grid search was employed to determine the partial optimal hyperparameters, which were overfitted by default; then, adjusted feature numbers were included in the ML model according to the fitting situation of model; finally, according to the above results, grid search was executed to find out the global optimal hyperparameters.

### Model explanation

Shapley additive explanations (SHAP) analysis is derived from a game theory concept and has the advantage of gaining insight into the association between observations and clinical outcome [16]. When the SHAP value of feature observation was greater than zero, its effect on model output was positive. This method can help increase visibility and interpretability of the ML model. In this study, SHAP analysis was used to explain the decision-making process of the ML model, including sorting the features by importance, showing the association between observed values and risk, determining cut-off points and exploring the contribution of significant outliers. Model development and explanation were conducted in Python, version 3.8.8; scikit-learn, version 0.24.2 and SHAP, version 0.40.0.

### Statistical analysis

Because the missing values for most laboratory examination and echocardiographic indexes were replaced with their medians, they were described as the median (IQR) and compared using Mann–Whitney U test. Continuous variables with normal distribution were expressed as the mean ± SD and compared by one-way ANOVA. Categorical data were described as numbers and percentages, and the Chi square test or Fisher’s exact test were used where appropriate. Receiver Operating Characteristic (ROC) curve, Precision-Recall curve and Decision Curve Analysis were used on the testing cohort to show the ability of ML model to classify correctly. Youden index was used to determine the threshold of each continuous covariate in the training cohort. Kaplan–Meier curve was used to describe time to AF recurrence. Univariable and multivariable Cox proportional hazard regression analyses were used to explore the potential risk factors. Risk ratio (RR) with 95% confidence interval (CI) was used to describe the potential risk factors. A two-sided *p* value < 0.05 was considered statistically significant. Statistical analyses were conducted on SPSS statistics 26.0 and R 4.2.0.

## Results

### Patients and baseline characteristics

A total of 1496 patients were screened in this study. Finally, 471 patients were enrolled. The median follow-up time was 25 months (IQR: 13–36 months). Patient follow-up totaled an aggregate of 954.3 patient-years with 135 patients (14.2/100 patient-years) experiencing tachycardias recurrences (Additional file 1: Fig. S1).

The entire cohort was divided based on the clinical outcome and 336 patients were assigned into the sinus rhythm maintenance group. Baseline characteristics are shown in Table 1. In total, the majority of demographic characteristics, all concomitant diseases and discharge medications did not show statistically significant differences between these two groups, excepted male sex (60.7% vs. 50.4%, *p* = 0.040), AF duration (24 months vs. 48 months, *p* = 0.002) and ERAF (1.8% vs. 31.9%, *p* < 0.001). Despite many patients having cardiac comorbidities, such as hypertension (42.0% and 45.2%, *p* = 0.52), the indicators corresponding to these diseases were relatively normal (e.g., systolic blood pressure, SBP, 125.5 ± 18.42 vs. 127.6 ± 17.8, *p* = 0.26). No statistical significance was detected in echocardiographic indexes and laboratory examinations before (Additional file 1: Table S1) and after (Table 1) median imputation.

**Table 1:** Patient characteristics at baseline

Characteristics	Sinus Rhythm n = 336	pxAF recurrence n = 135	Missing Data	*p* value
Age, years	59.9 ± 10.8	60.8 ± 10.5	0	0.42
Male, n (%)	204 (60.7%)	68 (50.4%)	0	0.05
BMI, kg/m^2^	25.0 ± 3.2	24.9 ± 3.2	0	0.66
DBP, mmHg	73.8 ± 12.7	74.5 ± 11.9	0	0.54
SBP, mmHg	125.5 ± 18.42	127.6 ± 17.8	0	0.26
Smoker, n (%)	142 (42.3%)	63 (46.7%)	0	0.41
CHA_2_DS_2_-VASc score	1.5 ± 1.3	1.69 ± 1.2	0	0.27
HASBLED score	1.8 ± 0.8	1.8 ± 0.7	0	0.96
AF duration, months	24.0 (12.0, 60.0)	48.0 (12.0, 96.0)	0	< 0.01**
Hypertension, n (%)	141 (42.0%)	61 (45.2%)	0	0.54
Coronary artery disease, n (%)	59 (17.6%)	21 (15.6%)	0	0.69
Type 2 diabetes, n (%)	39 (11.6%)	16 (11.9%)	0	1.00
Chronic heart Failure, n (%)	27 (8.0%)	9 (6.7%)	0	0.70
Atrial septal defect, n (%)	68 (20.2%)	29 (21.5%)	0	0.80
Left atrium diameter, mm	39.0 (36.0, 42.0)	40.0 (37.0, 43.0)	35 (7.4%)	0.06
Left ventricular ejection fraction, %	58.0 (55.0, 59.0)	58.0 (55.0, 60.0)	34 (7.2%)	0.36
Laboratory examination				
White blood cell count, × 10^9^/L	5.9 (5.0, 6.9)	5.9 (5.0, 7.0)	4 (0.8%)	0.75
Neutrophil to Lymphocyte ratio	2.2 (1.7, 2.9)	2.1 (1.7, 3.0)	4 (0.8%)	0.94
Total triglyceride, mmol/L	1.2 (0.9, 1.6)	1.3 (0.9, 1.7)	28 (5.9%)	0.84
HDL-C, mmol/L	1.1 (1.0, 1.3)	1.1 (1.0, 1.3)	41 (8.7%)	0.53
LDL-C, mmol/L	2.3 (1.7, 2.6)	2.2 (1.8, 2.7)	41 (8.7%)	0.42
NT-proBNP, pg/mL	168.6 (71.1, 457.4)	168.6 (82.5, 487.2)	52 (11.0%)	0.33
D-Dimer, ng/mL	0.2 (0.1, 0.3)	0.2 (0.1, 0.3)	18 (3.8%)	0.57
Radiofrequency ablation, n (%)	137 (40.8%)	62 (45.9%)	0	0.35
Discharge medication				
Propafenone, n (%)	195 (58.0%)	68 (50.4%)	0	0.15
Amiodarone/Dronedarone, n (%)	99 (29.5%)	43 (31.9%)	0	0.66
Beta receptor blocker, n (%)	56 (16.7%)	29 (21.5%)	0	0.23
RAAS inhibitor, n (%)	132 (39.3%)	54 (40.0%)	0	0.92
Statin, n (%)	47 (14.0%)	15 (11.1%)	0	0.45
ERAF, n (%)	6 (1.8%)	43 (31.9%)	0	< 0.01**

Data are mean ± SD, median (IQR) or n (%). Missing values were filled in with the median

CHA_2_DS_2_-VASc score is a system to estimate stroke and HASBLED score to estimate major bleeding in AF patients. AF duration indicates time since first AF diagnosis. BMI body mass index, DBP diastolic blood pressure, SBP systolic blood pressure, AF paroxysmal atrial fibrillation, RAAS renin–angiotensin–aldosterone system, ERAF early recurrence of atrial fibrillation. **p values < 0.01

### Model development

**Fig. 2:**
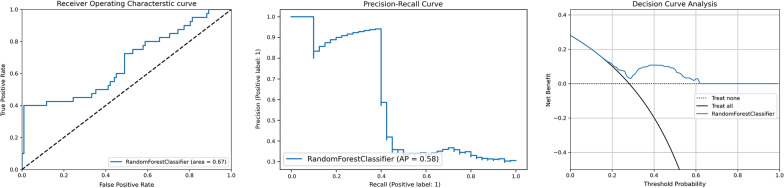
Model performance in testing cohort

Based on the 7:3 ratio, 329 and 142 patients were randomly assigned into the training and testing cohorts, respectively. Baseline characteristics are summarized in Table 2. Using the hyperparameters confirmed through tenfold cross validation, an accuracy of 0.786 in the training cohort was acquired, with the precision rate, recall rate, F1-score and area under the ROC curve (AUROC) being 0.964, 0.284, 0.439 and 0.640 respectively. Performance of the model in the testing cohort was robust. The accuracy, precision rate, recall rate, F1-score and AUROC were 0.803, 0.929, 0.325, 0.482 and 0.667, respectively. The hyperparameters used are shown in Additional file 1: Table S2 and model performance in the testing cohort is presented in Fig. 2.

**Table 2:** Baseline characteristics between training cohort and testing cohort

Characteristics	Training cohort n = 329	Testing cohort n = 142	*p* value
Age, years	59.7 ± 11.1	61.2 ± 9.7	0.17
Male, n (%)	194 (59.0)	78 (55.0)	0.42
BMI, kg/m^2^	25.0 ± 3.2	24.8 ± 3.2	0.50
Diastolic blood pressure, mmHg	73.5 ± 11.6	75.1 ± 14.3	0.18
Systolic blood pressure, mmHg	125.3 ± 18.7	127.9 ± 17.2	0.17
Smoker, n (%)	144 (43.8)	61 (43.0)	0.92
CHA_2_DS_2_-VASc score	1.5 ± 1.2	1.8 ± 1.4	0.02*
HASBLED score	1.7 ± 0.7	1.9 ± 0.8	0.03*
AF duration, months	24.0 (11.0, 60.0)	36.0 (12.0, 72.0)	0.44
Hypertension, n (%)	130 (39.5)	72 (50.7)	0.03*
Coronary artery disease, n (%)	50 (15.2)	30 (21.1)	0.14
Type 2 diabetes, n (%)	35 (10.6)	20 (14.1)	0.28
Chronic heart Failure, n (%)	24 (7.3)	12 (8.5)	0.71
Atrial septal defect, n (%)	59 (16.9)	38 (26.8)	0.04*
Left atrium diameter, mm	39.0 (36.0, 42.0)	39.0 (36.0, 42.3)	0.83
Left ventricular ejection fraction, %	58.0 (55.0, 60.0)	58.0 (55.0, 59.0)	0.96
Laboratory examination			
White blood cell count, × 10^9^/L	5.9 (5.0, 7.0)	5.9 (5.0, 6.8)	0.80
Neutrophil to Lymphocyte ratio	2.2 (1.7, 2.8)	2.4 (1.7, 3.1)	0.08
Total triglyceride, mmol/L	1.2 (0.9, 1.6)	1.2 (0.9, 1.6)	0.62
HDL-C, mmol/L	1.1 (1.0, 1.3)	1.1 (1.0, 1.3)	0.70
LDL-C, mmol/L	2.2 (1.7, 2.7)	2.2 (1.8, 2.6)	0.93
NT-proBNP, pg/mL	168.6 (73.5, 437.1)	168.6 (88.8, 535.6)	0.21
D-Dimer, ng/mL	0.2 (0.2, 0.3)	0.2 (0.1, 0.3)	0.19
Radiofrequency ablation, n (%)	136 (41.3)	63 (44.4)	0.54
Discharge medication			
Propafenone, n (%)	189 (57.4)	74 (52.1)	0.31
Amiodarone/Dronedarone, n (%)	94 (28.6)	48 (33.8)	0.28
Beta receptor blocker, n (%)	61 (18.5)	24 (16.9)	0.70
RAAS inhibitor, n (%)	127 (38.6)	59 (41.5)	0.61
Statin, n (%)	42 (12.8)	20 (14.1)	0.77
ERAF, n (%)	32 (9.7)	17 (12.0)	0.51

Data are mean ± SD, median (IQR) or n (%)

AF duration indicates time since first AF diagnosis. BMI body mass index, DBP diastolic blood pressure, SBP systolic blood pressure, AF paroxysmal atrial fibrillation, RAAS renin–angiotensin–aldosterone system, ERAF early recurrence of atrial fibrillation. *p values < 0.05

### SHAP analysis

**Fig. 3:**
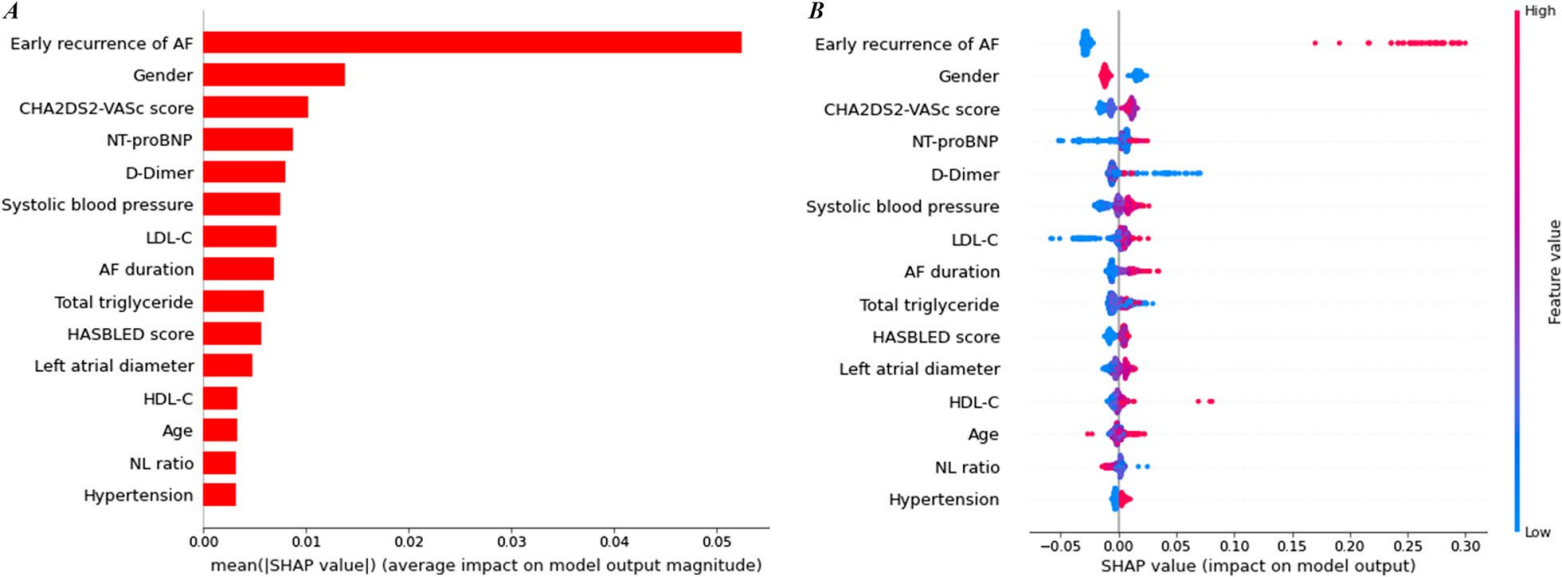
Summary plot for ML model. Summary plot **A** showed the top 15 features evaluated by mean absolute SHAP values. Summary plot **B**, patients in the whole cohort were described as different dots. The x-axis location of each dot represented the SHAP value of its corresponding feature at the same row. The colors of dots represented the values from small to large, or negative to positive of their corresponding observed values of features, which were reflected on the color gradient on the right from blue to red. AF atrial fibrillation, LDL-C low-density lipoprotein cholesterol, HDL-C high-density lipoprotein cholesterol, NL ratio neutrophil lymphocyte ratio

**Fig. 4:**
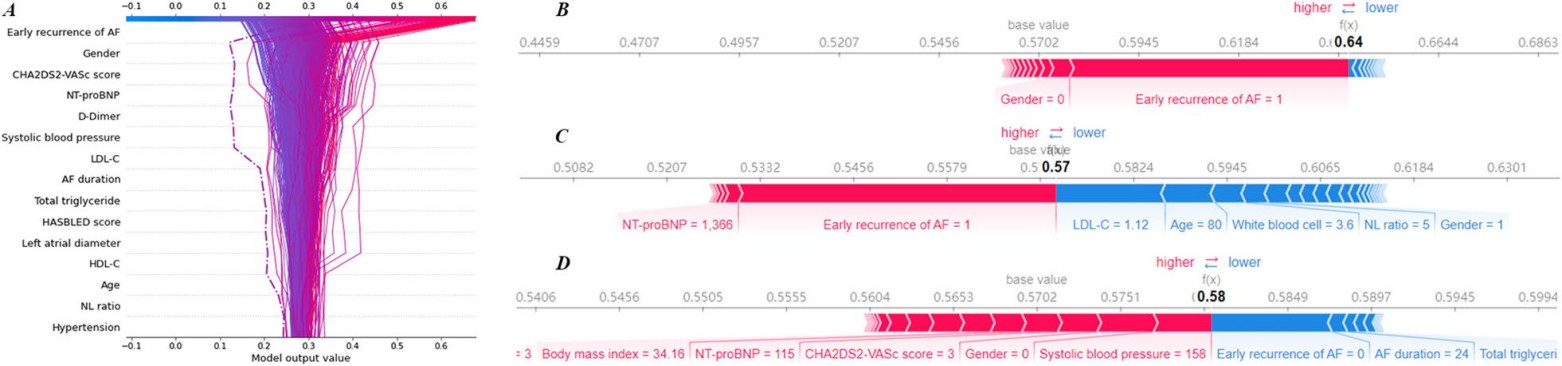
Decision plot and force plot for ML model. **A** Decision plot, patients in the whole cohort were described as different lines. The bottom-up variation of lines represented the influence of corresponding features on model output. **B–D** force plot intuitively revealed patients prediction. In (**A**), an outlier was marked with dashed line, and the corresponded patient could be seen in (**C**). AF atrial fibrillation, LDL-C low-density lipoprotein cholesterol, HDL-C high-density lipoprotein cholesterol, NL ratio neutrophil lymphocyte ratio

The top 15 features with descending importance ranked by mean absolute SHAP values are shown in the summary plot (Fig. 3A). Figure 3B shows the relationship between observations of the top 15 features and SHAP values: patients are represented by different dots, and the x-axis location of each dot is the SHAP value of its corresponding feature at the same row. For continuous variables, the dot color represents the values from small to large of their corresponding observed values of features, which are reflected on the color gradient on the right from blue to red. For categorial variables, the blue dots represent male sex or positive, and the red dots represent female sex or negative. ERAF showed the most positive impact on model output. Female patients had a higher risk of AF recurrence compared to male patients. The dots of these two features were completely distributed on both sides of the y-axis. Figure 4B shows the prediction process for a representative patient, and a true decision could be made through the above two features. Continuous variables, such as CHA_2_DS_2_-VASc score, NT-proBNP, SBP, AF duration, HAS-BLED score and left atrial diameter (LAD), showed positive impact on model output, and SHAP values increased as their observations increased. On the other hand, neutrophil lymphocyte ratio showed negative impact on model output, and SHAP values decreased as its observations decreased. The impact of D-Dimer and total triglycerides (TG) on outcome prediction could not be identified because there was no clear correspondence between their observed values and SHAP values.

**Fig. 5:**
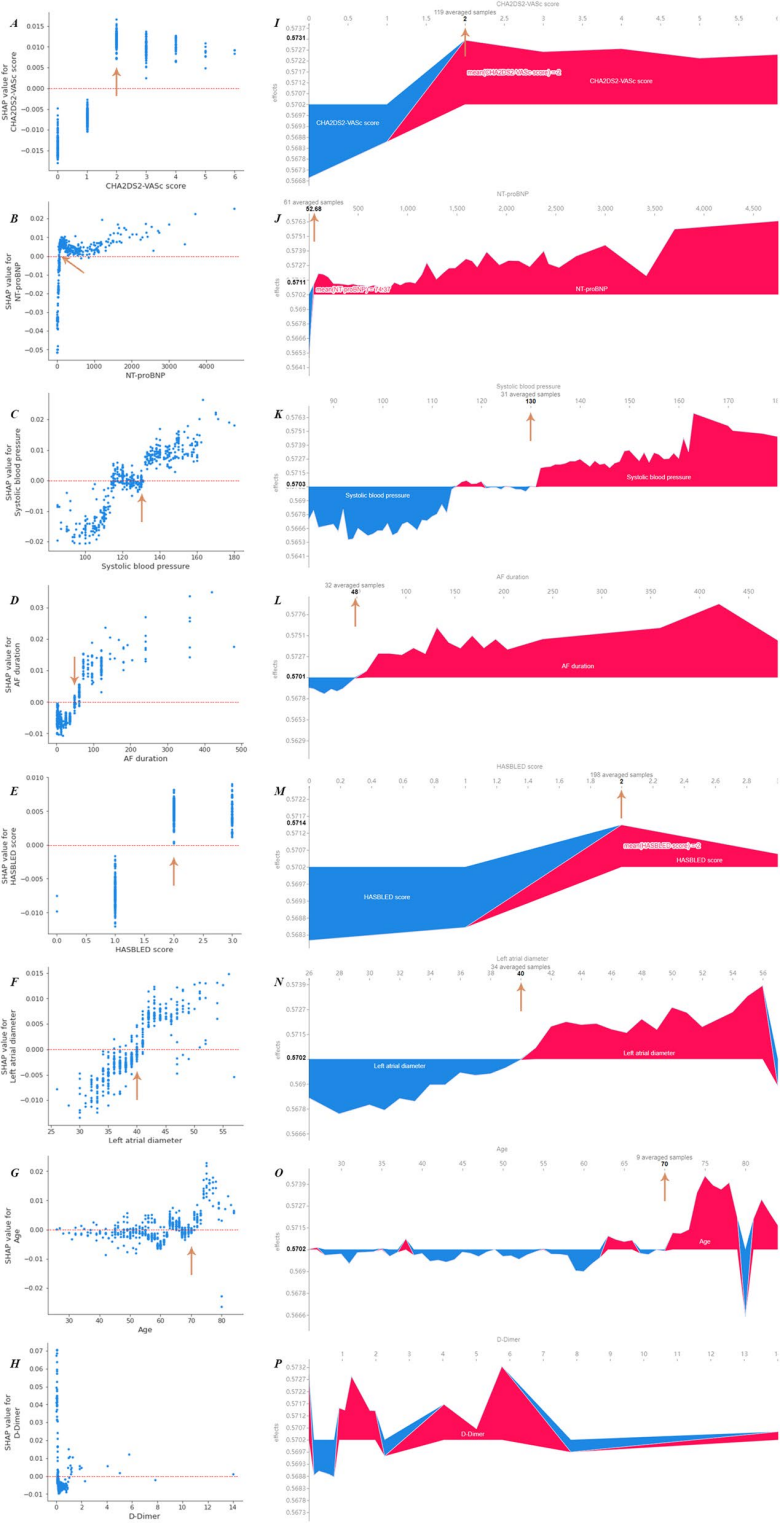
The impact of single feature on the outcome prediction. Dependence plots and force plots showed how single features influenced model output. Red dashed lines represented SHAP = 0. Thresholds were indicated by orange arrows. AF atrial fibrillation

SHAP dependence plots show the impact of a single feature on outcome prediction (Fig. 5A–H). The plots reveal the type of relationship between observed values and risk (i.e., linear, monotonous or complex). Three types of scatter distributions show: (i) roughly monotonous increasing type, continuous variables with positive impact on model output, except age, belong to this type; (ii) roughly symmetric distribution type. For example, data points for age were equally distributed on both sides of SHAP = 0, when the observations of age were less than 65; (iii) U-shaped, e.g. D-Dimer. For the first two types of distributions, the high-risk cut-off points were easily identified with the help of force plots (Fig. 5I–P): the thresholds of CHA_2_DS_2_-VASc score, SBP, AF duration, HAS-BLED score, LAD and age were 2, 130 mmHg, 48 months, 2, 40 mm and 70 years, respectively; the threshold of NT-proBNP was 52.28 pg/mL, which was lower than its upper limit of medical reference (125.00 pg/mL). Thresholds of other important features are shown in Additional file 1: Fig. S2.

For continuous variables, the influence of outliers on model output is visualized in a decision plot (Fig. 4A). Patients in the entire cohort are described as different lines. The bottom-up variation of lines represents the influence of corresponding features on outcome prediction. When the observed value of a feature is highly influential on model output, the corresponding curve will deviate from most curves significantly. Discovery and personal influence of outliers are shown in Fig. 4A, C, D.

### Statistical significance of thresholds

**Fig. 6:**
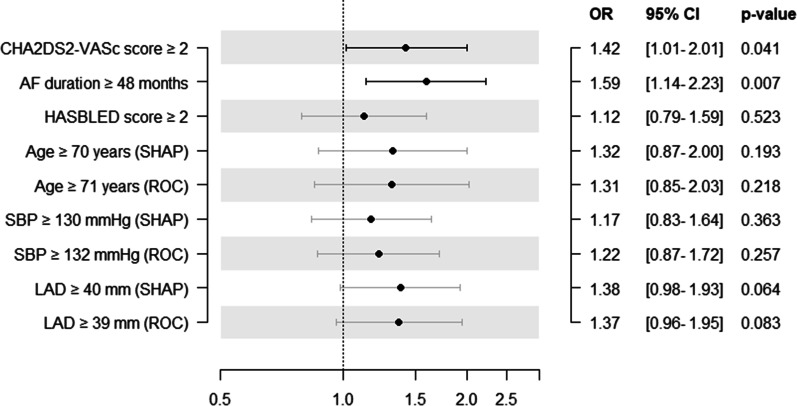
Univariable Cox proportional hazard regression based on high-risk thresholds. AF atrial fibrillation, SBP systolic blood pressure, LAD left atrial diameter

Statistical thresholds of continuous variables in the training cohort evaluated by Youden index were similar to those determined by SHAP analysis (Table 3). Univariable Cox proportional hazard regression analysis showed that only CHA_2_DS_2_-VASc score ≥ 2 and AF duration ≥ 48 months were statistically significant in the entire cohort (Fig. 6).

**Table 3:** Thresholds of continuous variables in training cohort based on Youden index

	Threshold	Sensitivity	1-Specificity	Youden index
Age, years	71	0.211	0.128	0.083
Systolic blood pressure, mmHg	132	0.432	0.321	0.111
CHA_2_DS_2_-VASc score	2	0.589	0.389	0.200
HASBLED score	2	0.674	0.556	0.118
AF duration, months	48	0.484	0.359	0.125
Left atrial diameter, mm	39	0.653	0.564	0.089

BMI body mass index, AF atrial fibrillation

## Discussion

In this study, an explainable ML model was developed and tested to reveal its decision-making process in identifying patients with paroxysmal AF at high risk for recurrence after catheter ablation. The top 15 features and their specific impact on outcome prediction were revealed through SHAP analysis. ERAF showed the most positive impact on model output, and female patients had a higher risk of AF recurrence compared to male patients. CHA_2_DS_2_-VASc score, NT-proBNP, SBP, AF duration, HAS-BLED score and LAD, showed positive impact on model output, and neutrophil lymphocyte ratio showed negative impact on model output. The effect of D-Dimer on model output was a U-shaped association. Furthermore, the explainable ML model was able to provide suitable thresholds of continuous variables and point out outliers. The thresholds of CHA_2_DS_2_-VASc score, SBP, AF duration, HAS-BLED score, LAD and age were 2, 130 mmHg, 48 months, 2, 40 mm and 70 years, respectively. SHAP analysis could show some errors in the ML model. These findings fully confirmed the role of the explainable ML model in post hoc analysis.

### Risk factors identification

Risk assessment for AF recurrence after catheter ablation remains a topic worth exploring. According to current guidelines, despite the fact that a series of prediction scores have been evaluated, their prediction ability is moderate; the most powerful predictor is ERAF [19]. SHAP analysis provides a new method to explore potential risk factors. The proposed model also revealed that ERAF was the most important feature for positive model output. Additionally, our model also considered demographic characteristics, underlying diseases and laboratory examination indexes. Their mean contributions are ranked in the summary plot (Fig. 3). Fahmy et al. revealed the association between late gadolinium enhancement cardiac magnetic resonance markers and the risks of cardiovascular hospitalization and all-cause death in non-ischemic cardiomyopathy by developing an explainable extreme gradient boosting model [20]. Their selected ML model was extreme gradient boosting, while the proposed one is RF. In fact, different ML models have different feature importance orders. Stenwig et al. revealed the decision-making process of four ML models including RF, logistic regression (LR), naïve Bayes (NB) and Adaptive Boosting (AdaBoost) in predicting hospital mortality in intensive care unit. Among them, RF, LR and NB revealed that the Glasgow coma Scale was the strongest feature on positive model output, but age was the strongest feature for AdaBoost [21]. More models could provide more perspective, while also making choices difficult. Until now, most previous studies have taken the approach of selecting the best performing model for visualization, while it was very difficult to develop a statistically significant ML model. As Stenwig et al. reported, the performances between RF, LR and AdaBoost were similar [21]. More reasonable evaluation strategies are warranted to increase the information available to physicians and aid in decision-making.

### Relationship between single feature and model output

SHAP analysis could illuminate the effect of every feature on the outcome from an objective perspective. CHA_2_DS_2_-VASc score was a risk factor; the SHAP values increased with the increase of its observed values. Neutrophil lymphocyte ratio was a protective factor; the SHAP values decreased with the increase of its observed values. The effect of D-Dimer on model output was a U-shaped association. The U-shaped association between variables and outcomes has been widely reported in previous studies. Serum uric acid (UA) level and in-hospital and 6-month mortality of infective endocarditis was shown to have a U-shaped association in a single-center cohort. After adjustment of a series of confounding variables, UA > 400 μmol/L or UA < 250 μmol/L were independent indicators of mortality. The risk of cardiovascular events in male patients with medium high-density lipoprotein cholesterol (HDL-C) was significantly decreased compared with male patients with low HDL-C (≤ 40 mg/dl) and male patients with high HDL-C (≥ 80 mg/dl), in which a non-linear U-shaped association evaluated by spline analysis was shown. In AF patients, a U-shaped association was observed between blood pressure and in-ICU or all-cause mortality, and patients with blood pressure was 110/55 mmHg had the lowest mortality [22–24]. This type of association was defined using multivariable regression combined with spline analysis. In the present study, SHAP analysis provided another method to define a U-shaped association. Compared with statistical methods, ML models do not require hypothesis testing; they only need to ensure the generalization ability in external testing cohorts. Additionally, these algorithms are non-parametric and are not affected by multi-collinearity between features. However, this approach has its own limitation: the U-shaped association in ML models was between feature observations and model output, rather than clinical outcomes. If ML models did not fit well, the results would be wrong. Therefore, pre-processing of features and determination of the optimal hyperparameters are crucial.

### Threshold determination

A threshold determines the score of each continuous variable in the clinical model, for example, age in CHA_2_DS_2_-VASc score [25]. However, thresholds of continuous variables are usually determined based to the Youden index, previous studies, or clinical experience. The proposed explainable ML model provided another method to confirm the thresholds of features which were evaluated by dependence plots combined with force plots. Numerically, thresholds determined by SHAP analysis were close to the statistical method results; in terms of results, the thresholds determined by the two methods were the same. Only CHA_2_DS_2_-VASc score ≥ 2 and AF duration ≥ 48 months could distinguish patients with high risk of AF recurrence. Nevertheless, thresholds determined by SHAP analysis were stable, results were same regardless of the cohort (i.e., training, testing, entire), while results of the statistical method were different between the three cohorts (Additional file 1: Table S3).

The thresholds of laboratory examination indexes evaluated by SHAP analysis were inaccurate. For example, the threshold of NT-proBNP was 52.28 pg/ml, which was less than the upper limit of regular medical reference. This was clearly an incorrect outcome. Stenwig et al. also reported that the decision-making process of LR did not correspond to common medical theory: a linear relationship was detected between temperature and mortality, with higher temperatures being associated with better prognosis [21]. This phenomenon explained why the results of conventional ML models are sometimes unacceptable. While with the help of SHAP analysis, physicians can easily adopt their correct prediction and make more accurate decisions based on their own clinical experience.

### Model performance in high-dimensional data

Multivariable Cox regression results showed that ERAF, AF duration and left ventricular ejection fraction were statistically significant, with an AUROC value of 0.513 (Additional file 1: Table S4 and S5). On one hand, traditional statistical models comprised features with statistical significance. On the other hand, many contributory outliers were removed when constructing the statistical model. This could also be seen from Fig. 5C that an SBP of 158 mmHg contributed more to predict AF recurrence than female sex. The above-mentioned reasons may lead to statistical model under-fitting.

Currently, there are conflicting reports as to whether ML models perform better than traditional statistical methods. Moncada et al. [26] showed that an extreme gradient boosting model performed considerably better than a Cox model in the prediction of breast cancer survival in a large database. On the other hand, two large-scale studies showed similar performances between ML models and the routine statistical method. Loring et al. indicated that stepwise multivariable logistic statistics regression model performed as well or better than ML models with specific hyperparameters in the prediction of death, major bleeding and stroke in two large AF cohorts [27]. The performance of ML model in predicting long-term risk maybe limited in terms of survival analysis [28]. However, large-scale data does not mean high-dimensionality data. The former emphasizes data size, while the latter the number of features. In the presence of too many features, statistical methods will be overloaded. It is postulated that compared to statistical methods, ML performs better in handling high-dimensionality data.

Recently, three-dimensional data, from computer tomography or magnetic resonance images for example, have already been used in automatic feature selection by DL and risk stratification models have been built based on the selected features, to identify patients at high risk [9–12]. These features, especially radiomic features, effectively compensated for the deficiency of current risk factors for AF recurrence. In general, radiomics methods can provide hundreds or thousands of obscure features, and only explainable AI algorithms can effectively comprehend and visualize them. In summary, to the authors believe that AI-based risk assessment after AF ablation can be widely used in clinical and daily life in the same way as AI-based early identification of AF [29, 30].

### Explainable ML model in clinical practice

Explainable ML model can make clinical practice more accurate. When the characteristics of each patient are entered into the model, the model first predicts whether he or she is at high risk. After that, we can understand the decision-making process of the model through SHAP analysis. According to the weight of different features and thresholds of features, physicians are also able to make rational clinical decisions. If there is any error in decision-making process, physicians can clearly see the unreasonable judgements in model prediction, to combine model outputs and clinical experience to further confirm whether patient is at high risk. Besides, with the increase of sample size in the training cohort of model, the accuracy of the model will also be improved. Perhaps human–computer interaction will become a trend in clinical practice in the future.

### Limitations

There were several limitations in this study. The sample size in our study was small. Median values were used to fill in the missing data for laboratory examinations and echocardiographic indicators, which may reduce power of test and representativeness. Potential risk factors proposed by other studies such as abnormal estimated glomerular filtration rate, and chronic obstructive pulmonary disease were not considered because of insufficient confirmed cases [4, 31]. This made the proposed ML model incomparable with previous risk scores. In terms of methodology, an ML model with time-to-event type analysis called Random Survival Forests was not adopted because its visualized analysis was not available at that time. Consequently, RF was employed instead. Previous studies often used AUROC as the evaluation index for adjusting hyperparameters, while here, this led to severe overfitting. The AUROCs were 0.940 and 0.551 in the training and testing cohorts, respectively. Considering the presence of a large number of positive samples in the cohort, accuracy was chosen as the index for hyperparameter adjustment. The temporal, spatial and human distribution of this retrospective cohort was relatively homogeneous. There was no external validation, and splitting of population to 7:3 or 9:1 consisted only of internal validation, which may limit the generalizability of the ML model and affect the accuracy of results.

## Conclusions

This study showed that the explainable machine learning model can effectively reveal its decision-making process in identifying patients with paroxysmal atrial fibrillation at high risk for recurrence after catheter ablation by listing important features, showing the impact of every feature on model output, determining appropriate thresholds and identifying significant outliers. Physicians could clearly see the unreasonable judgements in model prediction, to combine model outputs and clinical experience and assist in decision-making. Studies based on high-dimensional databases with large sample size are necessary to further confirm the universality of this finding.

## Supplementary Information

**Additional file 1**. Supplementary Material.

## Data Availability

The datasets used and/or analyzed during the current study can be available from the corresponding author on reasonable request.

## Abbreviations

AdaBoost: Adaptive boosting
AF: Atrial fibrillation
AUC: Area under the receiver operating characteristic curve
BP: Blanking period
CI: Confidence interval
DL: Deep learning
DT: Decision tree
ERAF: Early recurrence of atrial fibrillation
HDL-C: High-density lipoprotein cholesterol
LAD: Left atrial diameter
LR: Logistic regression
ML: Machine learning
NB: Naïve bayes
RF: Random forest
RR: Risk ratio
ROC: Receiver operating characteristic
SBP: Systolic blood pressure
SHAP: Shapley additive explanations
TG: Total triglycerides
TRIPOD: Transparent reporting of a multivariable prediction model for individual prognosis or diagnosis
UA: Uric acid

## References

[1] Lesh MD, Diederich C, Guerra PG (1999). An anatomic approach to prevention of atrial fibrillation: pulmonary vein isolation with through-the-balloon ultrasound ablation (TTB-USA). Thorac Cardiovasc Surg.

[2] Haïssaguerre M, Jaïs P, Shah DC (1998). Spontaneous initiation of atrial fibrillation by ectopic beats originating in the pulmonary veins. N Engl J Med.

[3] Imberti JF, Ding WY, Kotalczyk A (2021). Catheter ablation as first-line treatment for paroxysmal atrial fibrillation: a systematic review and meta-analysis. Heart.

[4] de Vos CB, Pisters R, Nieuwlaat R (2010). Progression from paroxysmal to persistent atrial fibrillation clinical correlates and prognosis. J Am Coll Cardiol.

[5] Kornej J, Hindricks G, Shoemaker MB (2015). The APPLE score: a novel and simple score for the prediction of rhythm outcomes after catheter ablation of atrial fibrillation. Clin Res Cardiol.

[6] Winkle RA, Jarman JW, Mead RH (2016). Predicting atrial fibrillation ablation outcome: the CAAP-AF score. Heart Rhythm.

[7] Wang G, Zhang Y, Li S (2021). A machine learning-based prediction model for cardiovascular risk in women with preeclampsia. Front Cardiovasc Med.

[8] Budzianowski J, Hiczkiewicz J, Burchardt P (2019). Predictors of atrial fibrillation early recurrence following cryoballoon ablation of pulmonary veins using statistical assessment and machine learning algorithms. Heart Vessels.

[9] Shade JK, Ali RL, Basile D (2020). Preprocedure application of machine learning and mechanistic simulations predicts likelihood of paroxysmal atrial fibrillation recurrence following pulmonary vein isolation. Circ Arrhythm Electrophysiol.

[10] Liu CM, Chang SL, Chen HH (2020). The clinical application of the deep learning technique for predicting trigger origins in patients with paroxysmal atrial fibrillation with catheter ablation. Circ Arrhythm Electrophysiol.

[11] Firouznia M, Feeny AK, LaBarbera MA (2021). Machine learning-derived fractal features of shape and texture of the left atrium and pulmonary veins from cardiac computed tomography scans are associated with risk of recurrence of atrial fibrillation postablation. Circ Arrhythm Electrophysiol.

[12] Atta-Fosu T, LaBarbera M, Ghose S (2021). A new machine learning approach for predicting likelihood of recurrence following ablation for atrial fibrillation from CT. BMC Med Imaging.

[13] Yang M, Cao Q, Xu Z (2022). Development and validation of a machine learning-based radiomics model on cardiac computed tomography of epicardial adipose tissue in predicting characteristics and recurrence of atrial fibrillation. Front Cardiovasc Med.

[14] Roney CH, Sim I, Yu J (2022). Predicting atrial fibrillation recurrence by combining population data and virtual cohorts of patient-specific left atrial models. Circ Arrhythm Electrophysiol.

[15] Nuñez-Garcia JC, Sánchez-Puente A, Sampedro-Gómez J (2022). Outcome analysis in elective electrical cardioversion of atrial fibrillation patients: development and validation of a machine learning prognostic model. J Clin Med.

[16] Lundberg SM, Lee S-I. A unified approach to interpreting model predictions. Adv Neural Inf Process Syst. 2017;4765–74.

[17] Kuck KH, Brugada J, Fürnkranz A (2016). Cryoballoon or radiofrequency ablation for paroxysmal atrial fibrillation. N Engl J Med.

[18] Petch J, Di S, Nelson W (2022). Opening the black box: the promise and limitations of explainable machine learning in cardiology. Can J Cardiol.

[19] Hindricks G, Potpara T, Dagres N (2021). 2020 ESC Guidelines for the diagnosis and management of atrial fibrillation developed in collaboration with the European association for cardio-thoracic surgery (EACTS). Eur Heart J.

[20] Fahmy AS, Csecs I, Arafati A (2022). An explainable machine learning approach reveals prognostic significance of right ventricular dysfunction in nonischemic cardiomyopathy. JACC Cardiovasc Imag.

[21] Stenwig E, Salvi G, Rossi PS (2022). Comparative analysis of explainable machine learning prediction models for hospital mortality. BMC Med Res Methodol.

[22] Wei X, Fu B, Chen X (2021). U-shaped association between serum uric acid and short-term mortality in patients with infective endocarditis. Front Endocrinol.

[23] Trimarco V, Izzo R, Morisco C (2022). High HDL (high-density lipoprotein) cholesterol increases cardiovascular risk in hypertensive patients. Hypertension.

[24] Shao Y, Hu J (2022). U-shaped association between blood pressure and mortality risk in ICU patients with atrial fibrillation: the MIMIC-III database. Front Cardiovasc Med.

[25] Lip GY, Nieuwlaat R, Pisters R (2010). Refining clinical risk stratification for predicting stroke and thromboembolism in atrial fibrillation using a novel risk factor-based approach: the euro heart survey on atrial fibrillation. Chest.

[26] Moncada-Torres A, van Maaren MC, Hendriks MP (2021). Explainable machine learning can outperform Cox regression predictions and provide insights in breast cancer survival. Sci Rep.

[27] Loring Z, Mehrotra S, Piccini JP (2020). Machine learning does not improve upon traditional regression in predicting outcomes in atrial fibrillation: an analysis of the ORBIT-AF and GARFIELD-AF registries. Europace.

[28] Li Y, Sperrin M, Ashcroft DM (2020). Consistency of variety of machine learning and statistical models in predicting clinical risks of individual patients: longitudinal cohort study using cardiovascular disease as exemplar. BMJ.

[29] Attia ZI, Noseworthy PA, Lopez-Jimenez F (2019). An artificial intelligence-enabled ECG algorithm for the identification of patients with atrial fibrillation during sinus rhythm: a retrospective analysis of outcome prediction. Lancet.

[30] Perez MV, Mahaffey KW, Hedlin H (2019). Large-scale assessment of a smartwatch to identify atrial fibrillation. N Engl J Med.

[31] van der Burgh AC, Geurts S, Ikram MA (2022). Bidirectional association between kidney function and atrial fibrillation: a population-based cohort study. J Am Heart Assoc.

