# Supplementary material for: Explainable machine learning model reveals its decision-making process in identifying patients with paroxysmal atrial fibrillation at high risk for recurrence after catheter ablation
Source: BMC Cardiovasc Disord. 2023 Feb 17;23:91. doi: 10.1186/s12872-023-03087-0 (PMC9936738; doi:10.1186/s12872-023-03087-0)
Supplement: Supplementary file 1 — Additional file 1. Supplementary Material. [file 12872_2023_3087_MOESM1_ESM.docx]

**Supplementary Tables**

**Supplementary Table 1. Baseline characteristics without missing value imputation**

Data are mean ± SD, median (IQR) or n (%).

* BMI denotes body mass index, AF denotes paroxysmal atrial fibrillation, RAAS denotes renin-angiotensin-aldosterone system, ERAF denotes early recurrence of atrial fibrillation.

**Supplementary Table 2. The name of hyperparameters and their appropriate values**

Hyperparameter “random_state” was defined as custom.

**Supplementary Table 3. Thresholds determination**

AF indicates atrial fibrillation.

**Supplementary Table 4. Univariable Cox proportional hazard regression in training cohort**

* BMI denotes body mass index, AF denotes paroxysmal atrial fibrillation, RAAS denotes renin-angiotensin-aldosterone system, ERAF denotes early recurrence of atrial fibrillation.

**Supplementary Table 5. Multivariable Cox proportional hazard regression in training cohort**

* AF denotes paroxysmal atrial fibrillation, ERAF denotes early recurrence of atrial fibrillation.

**Supplementary figures and figure legends**

**Supplementary Fig. 1. Time to AF recurrence**

**Supplementary Fig. 2. Thresholds of another four features**

* LDL-C indicates low-density lipoprotein cholesterol, HDL-C indicates high-density lipoprotein cholesterol, NL ratio indicates neutrophil lymphocyte ratio.

**Supplementary Table 1. Baseline characteristics without missing value imputation**

| Characteristics | Sinus Rhythm  n = 336 | pxAF recurrence  n = 135 | Missing  Data | *p*-value |
| --- | --- | --- | --- | --- |
| Age, years | 59.9 ± 10.8 | 60.8 ± 10.5 | 0 | 0.42 |
| Male, n (%) | 204 (60.7%) | 68 (50.4%) | 0 | 0.05 |
| BMI, kg/m^2^ | 25.0 ± 3.2 | 24.9 ± 3.2 | 0 | 0.66 |
| Diastolic blood pressure, mmHg | 73.8 ± 12.7 | 74.5 ± 11.9 | 0 | 0.54 |
| Systolic blood pressure, mmHg | 125.5 ± 18.42 | 127.6 ± 17.8 | 0 | 0.26 |
| Smoker, n (%) | 142 (42.3%) | 63 (46.7%) | 0 | 0.41 |
| CHA_2_DS_2_-VASc score | 1.5 ± 1.3 | 1.69 ± 1.2 | 0 | 0.27 |
| HASBLED score | 1.8 ± 0.8 | 1.8 ± 0.7 | 0 | 0.96 |
| AF duration, months | 24.0 (12.0, 60.0) | 48.0 (12.0, 96.0) | 0 | < 0.01 |
| Hypertension, n (%) | 141 (42.0%) | 61 (45.2%) | 0 | 0.54 |
| Coronary artery disease, n (%) | 59 (17.6%) | 21 (15.6%) | 0 | 0.69 |
| Type 2 diabetes, n (%) | 39 (11.6%) | 16 (11.9%) | 0 | 1.00 |
| Chronic heart Failure , n (%) | 27 (8.0%) | 9 (6.7%) | 0 | 0.70 |
| Atrial septal defect , n (%) | 68 (20.2%) | 29 (21.5%) | 0 | 0.80 |
| Left atrium diameter, mm | 39.0 (36.0, 42.0) | 40.0 (37.0, 43.0) | 35 (7.4%) | 0.09 |
| Left ventricular ejection fraction, % | 57.0 (55.0, 60.0) | 58.0 (55.0, 60.0) | 34 (7.2%) | 0.38 |
| Laboratory examination  White blood cell count, ×10^9^/L  Neutrophil to Lymphocyte ratio  Total triglyceride, mmol/L  HDL-C, mmol/L  LDL-C, mmol/L  NT-proBNP, pg/mL  D-Dimer, ng/mL | 5.9 (5.0, 6.9)  2.2 (1.7, 2.9)  1.2 (0.9, 1.6)  1.1 (1.0, 1.3)  2.1 (1.7, 2.8)  159.7 (62.1, 506.8)  0.2 (0.1, 0.3) | 5.9 (5.0, 7.0)  2.1 (1.7, 3.0)  1.2 (0.9, 1.7)  1.1 (1.0, 1.3)  2.2 (1.7, 2.7)  183.6 (77.4, 511.7)  0.2 (0.1, 0.3) | 4 (0.8%)  4 (0.8%)  28 (5.9%)  41 (8.7%)  41 (8.7%)  52 (11.0%)  18 (3.8%) | 0.75  0.94  0.84  0.52  0.49  0.31  0.56 |
| Radiofrequency ablation, n (%) | 137 (40.8%) | 62 (45.9%) | 0 | 0.35 |
| Discharge medication  Propafenone, n (%)  Amiodarone/Dronedarone, n (%)  Beta receptor blocker, n (%)  RAAS inhibitor, n (%)  Statin, n (%) | 195 (58.0%)  99 (29.5%)  56 (16. 7%)  132 (39.3%)  47 (14.0%) | 68 (50.4%)  43 (31.9%)  29 (21.5%)  54 (40.0%)  15 (11.1%) | 0  0  0  0  0 | 0.15  0.66  0.23  0.92  0.45 |
| ERAF, n (%) | 6 (1.8%) | 43 (31.9%) | 0 | < 0.01 |

**Supplementary Table 2. The name of hyperparameters and their appropriate values**

| Name | Range | Step size | Appropriate value |
| --- | --- | --- | --- |
| n_estimators | 1 to 251 | 1 | 123 |
| max_depth | 1 to 30 | 1 | 3 |
| min_samples_leaf | 1 to 21 | 1 | 1 |
| min_samples_split | 2 to 42 | 1 | 2 |
| max_features | 1 to 31 | 1 | 5 |
| criterion | gini or entropy | – | gini |
| random_state* | 30 | – | 30 |

**Supplementary Table 3. Thresholds determination**

|  | SHAP analysis | | | Statistical method | | |
| --- | --- | --- | --- | --- | --- | --- |
|  | Whole  cohort | Training  cohort | Testing  cohort | Whole  cohort | Training  cohort | Testing  cohort |
| Age, years | 70 | 70 | 70 | 74 | 71 | 54 |
| Systolic blood pressure, mmHg | 130 | 130 | 130 | 112 | 132 | 145 |
| CHA_2_DS_2_-VASc score | 2 | 2 | 2 | 2 | 2 | 5 |
| HASBLED score | 2 | 2 | 2 | 2 | 2 | – |
| AF duration, months | 48 | 48 | 48 | 24 | 48 | 36 |
| Left atrial diameter, mm | 40 | 40 | 40 | 40 | 39 | 45 |

**Supplementary Table 4. Univariable Cox proportional hazard regression in training cohort**

|  | B | Wald | RR (95%CI) | *p*-value |
| --- | --- | --- | --- | --- |
| Age per 1 year | 0.007 | 0.494 | 1.007 (0.998, 1.025) | 0.482 |
| Female | 0.591 | 8.235 | 1.806 (1.206, 2.704) | 0.004 |
| BMI per 1 kg/m^2^ | 0.004 | 0.017 | 1.004 (0.944, 1.068) | 0.896 |
| Diastolic blood pressure per 1 mmHg | 0.002 | 0.047 | 1.002 (0.985, 1.019) | 0.829 |
| Systolic blood pressure per 1 mmHg | 0.008 | 2.028 | 1.008 (0.997, 1.018) | 0.154 |
| Smoker | 0.048 | 0.055 | 1.050 (0.701, 1.572) | 0.815 |
| CHA_2_DS_2_-VASc score per 1 | 0.170 | 5.061 | 1.185 (1.022, 1.374) | 0.024 |
| HASBLED score per 1 | 0.189 | 1.857 | 1.208 (0.920, 1.587) | 0.173 |
| AF duration per 1 months | 0.002 | 2.646 | 1.002 (1.000, 1.005) | 0.104 |
| Hypertension | 0.346 | 2.832 | 1.414 (0.945, 2.116) | 0.092 |
| Coronary artery disease | -0.140 | 0.220 | 0.869 (0.484, 1.561) | 0.639 |
| Type 2 diabetes | 0.147 | 0.209 | 1.158 (0.617, 2.172) | 0.648 |
| Chronic heart Failure | -0.034 | 0.007 | 0.967 (0.447, 2.088) | 0.931 |
| Atrial septal defect | -0.325 | 1.258 | 0.723 (0.409, 1.275) | 0.262 |
| Left atrium diameter per 1 mm | 0.026 | 1.658 | 1.026 (0.986, 1.068) | 0.198 |
| Left ventricular ejection fraction per 1% | 0.028 | 1.120 | 1.028 (0.976, 1.083) | 0.290 |
| White blood cell count per 1×10^-9^/L | -0.016 | 0.069 | 0.984 (0.871, 1.111) | 0.792 |
| Neutrophil to Lymphocyte ratio per 1 | 0.013 | 0.026 | 1.013 (0.869, 1.179) | 0.872 |
| Total triglyceride per 1 mmol/L | 0.069 | 0.482 | 1.072 (0.881, 1.304) | 0.488 |
| HDL-C per 1 mmol/L | 0.219 | 0.347 | 1.245 (0.601, 2.576) | 0.556 |
| LDL-C per 1 mmol/L | 0.176 | 1.790 | 1.192 (0.922, 1.542) | 0.181 |
| NT-proBNP per 1 pg/mL | 0.000 | 0.170 | 1.000 (1.000, 1.000) | 0.680 |
| D-Dimer per 1 ng/mL | 0.010 | 0.004 | 1.010 (0.720, 1.419) | 0.952 |
| Cryoballoon ablation | -0.133 | 0.408 | 0.876 (0.583, 1.315) | 0.523 |
| Propafenone | -0.187 | 0.824 | 0.829 (0.553, 1.243) | 0.364 |
| Amiodarone/Dronedarone | 0.086 | 0.147 | 1.090 (0.703, 1.690) | 0.701 |
| Beta receptor blocker | 0.370 | 2.311 | 1.448 (0.898, 2.335) | 0.128 |
| RAAS inhibitor | 0.125 | 0.358 | 1.133 (0.752, 1.707) | 0.550 |
| Statin | -0.204 | 0.403 | 0.816 (0.435, 1.531) | 0.526 |
| ERAF | 2.467 | 98.450 | 11.789 (7.241, 19.192) | 0.000 |

**Supplementary Table 5. Multivariable Cox proportional hazard regression in training cohort**

|  | B | Wald | RR | 95%CI | *p*-value |
| --- | --- | --- | --- | --- | --- |
| Step 1 | | | | | |
| ERAF | 2.637 | 161.810 | 13.971 | 9.306, 20.974 | 0.000 |
| Step 2 | | | | | |
| Left ventricular ejection fraction | 0.061 | 7.227 | 1.063 | 1.017, 1.112 | 0.007 |
| ERAF | 2.740 | 166.587 | 15.487 | 10.215, 23.478 | 0.000 |
| Step 3 | | | | | |
| AF duration | 0.003 | 6.193 | 1.003 | 1.001, 1.005 | 0.013 |
| Left ventricular ejection fraction | 0.060 | 6.812 | 1.061 | 1.015, 1.110 | 0.009 |
| ERAF | 2.712 | 160.093 | 15.060 | 9.894, 22.924 | 0.000 |


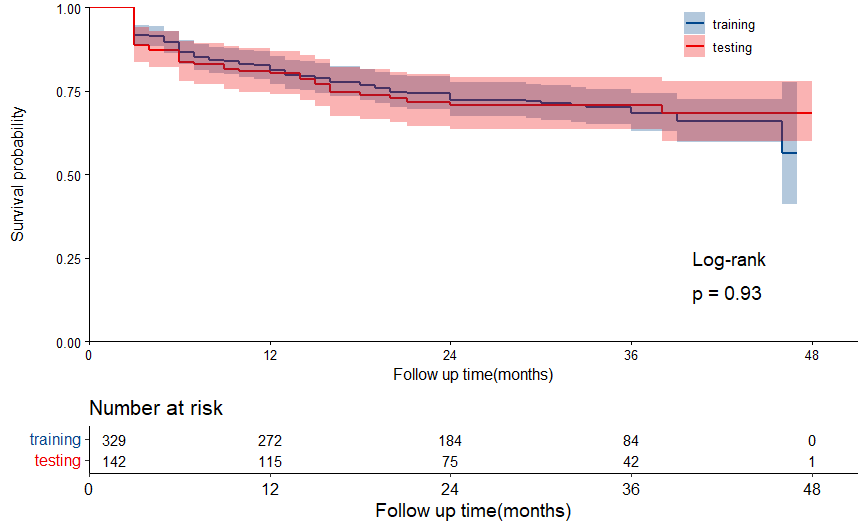


**Supplementary Fig. 1. Time to AF recurrence**

**
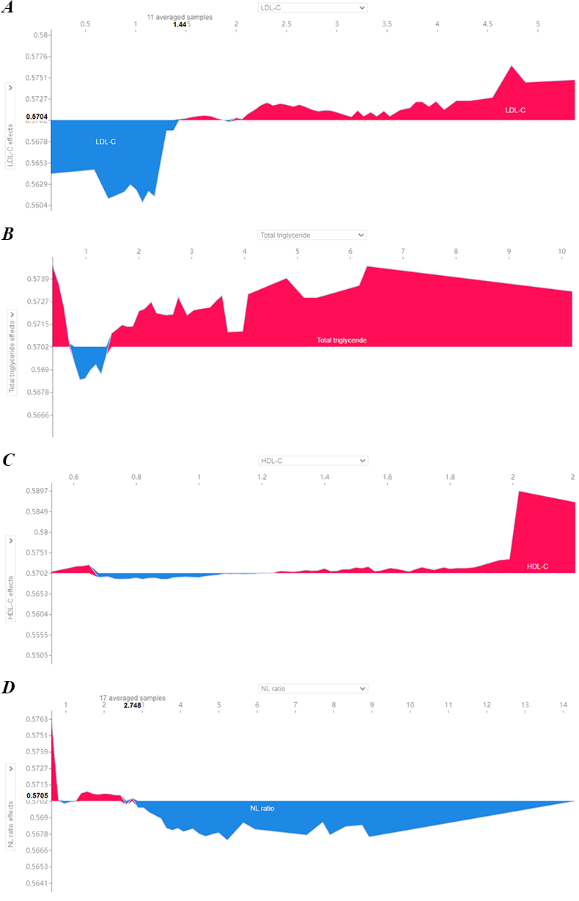
**

**Supplementary Fig. 2. Thresholds of another four features**
